# Experiences of African Students in Predominantly White Institutions: A Literature Overview

**DOI:** 10.1155/2016/5703015

**Published:** 2016-07-20

**Authors:** Davis Inyama, Allison Williams, Kay McCauley

**Affiliations:** ^1^Monash Health, Clayton, VIC 3168, Australia; ^2^School of Nursing and Midwifery, Monash University, Frankston, VIC 3199, Australia

## Abstract

The objective of this paper is to examine research conducted on the experiences of African health sciences students in predominantly white higher education institutions/environments. The main elements of cross-cultural adaptation models were adopted to discuss the amalgamated themes under the auspices of adjustment, integration, and conditioning. The overview revealed that African students encounter unique experiences, with isolation and “feeling different” being commonly mentioned. Recommendations for future research are presented, including programmatic implications for higher education and student affairs professionals.

## 1. Introduction

Experiencing life in developed countries such as the United States of America, United Kingdom, Canada, and Australia is often an aspirational dream for many people born in developing countries. Industrialization, affordable air travel, and the emergence of the computer age have aided this situation by reducing the world to a “small village” where people are mobile and traditional borders are more flexible. Education is one sector of the developed economies that has been heavily influenced by transborder movement with a considerable number of students coming from Africa.

According to Beoku-Betts [1], attitudes exist in many (developed) host nations which are linked to the widely held view that Africa is marginalized in the global system, a factor that greatly influences the experiences of black African students in predominantly white institutions. The students' experiences have often been attributed to existing stereotypes in the dominant culture about the people of the said cultural background [2]. Although various researchers have attempted to uncover the experiences of students studying in other countries, the experiences of black African students in predominantly white environments have not been fully explored, particularly those studies involving clinical placement. Clinical placement is defined as a rotation to which students are assigned in order to learn skills essential in the delivery of patient care. An African student in the context of this paper refers to a “black” university attendee who was born in Africa and shares and subscribes to given African beliefs, culture, or upbringing.

This paper aims to explore the literature deemed relevant to the experiences of African students, often classified as a minority, requiring clinical placement in predominantly white environments.

## 2. Method

### 2.1. Search Strategy

To examine what is known about black African health sciences students' experiences as they undertake clinical placement within healthcare organizations, an electronic search for literature mainly published between 1990 and 2015 was conducted using CINAHL, Medline, Google Scholar, and Scopus databases. In doing so, a combination of the following keywords was used: experiences, minority students, African students, African health sciences students, African nursing students, learning experience, and clinical experience. To expand the information catchment area, searches of websites, conference papers, and bibliographies of journal articles were also conducted.

Overall, 23 research papers were found to be relevant, although significant overlaps in findings were noted. The overview took a general course to first establish what existed in sources on African student experiences and later narrowed down to African health sciences students. The articles chosen were those that provided information on the experiences of minority students from all the health science disciplines in different countries and settings. Consideration was also given to methodological approaches used in the studies so as to establish existing differences in results and their appropriateness in answering the research questions in the respective studies (Table 1).

## 3. Theory

To explore the underlying phenomenology with regard to adapting to unfamiliar environments, cross-cultural adaptation models were used as frames of reference. While there are a number of models on cross-cultural adaptation such as the recuperation model [13], these models have similar features and principles pertaining to adjustment, learning, relations, interactions, and development in new environments [13]. The key components outlined in the various models were therefore amalgamated and modified to provide a framework in reviewing the research on experiences of the minority students, in particular black African, on the fronts of adjustment, integration, and conditioning.

### 3.1. Adjustment to New Environments

Adjustment occurs in response to a new work procedure, a new language or monetary system, a new social group, or a new world [14]. As a student, one must go through the adjustment phase, both socially and intellectually when transitioning into college life. Research has shown that the adjustment phase comes with various challenges and opportunities for the students. According to Anderson [13], elements essential in the process of adjustment are the presence of a motive, movement towards a given goal, and the presence of an obstacle. This is applicable, especially to the minority students as they have to adjust to environments where they are underrepresented [15].

### 3.2. Integrating

In order to be successful in a given environment, it is imperative that one fits into the norms and way of doing things in that particular society [14]. It is perhaps for this reason that student success has been linked to how well an individual integrates into the learning environment, particularly for minority students due to their small number [16].

### 3.3. Conditioning

Becoming part of a system requires an understanding on how to cope or react when faced with a given scenario. As part of being new members in the various institutions minority students have had to transform their behaviours in an attempt to cope. The journey through conditioning has positive and negative aspects. Summarily, it is worth noting that the level of adaptation is individually based and how different people respond to experiences during the adaptive process varies from person to person [13].

## 4. Highlights of the Overview

In keeping up with the purpose of the overview, the chosen studies were appraised and the standout aspects reported under the themes of adjustment, integrating, and conditioning.

### 4.1. Adjustment to New Environments

Several studies exploring the experiences of minority students have made contributions to the spectrum of student adjustment to their new learning environments [17, 18]. While results showed that some minority students easily adapted to their new earning environments, most pointed to several adjustment challenges that the students face in transitioning into their new environments [19].

According to Boafo-Arthur [3], many commencing students experience feelings of alienation and isolation as would generally be the case when adjusting to life and the culture present at a new educational institution. Boafo-Arthur [3] however revealed that black African international students further underwent discriminatory and prejudicial treatment that predisposed them to stress related to acculturation. The above findings were corroborated in a study conducted by Mwaura [9] with the aim of investigating the adjustment experiences of mature-age black African international students attending mainly white higher education colleges in the United States.

A qualitative study conducted by Constantine et al. [4] exploring the cultural adjustment experiences of international African students at an American college provided findings that also concurred with what had been advanced by Boafo-Arthur [3] and Mwaura [9]. Constantine et al. [4] interviewed twelve students from Kenya, Ghana, and Nigeria and reported how the students had to adjust their thinking to the reality of being in a new environment. From the study, it was revealed that the student's perception of America as the “perfect place” did not fall in line with what the students found out on arrival [4]. According to Constantine et al. [4], the truths and the harsh realities of the world the students were now in became evident thereby leading to the need to adjust their expectations. The study also reported that students had to adjust, from an environment where they were accepted, to the realities of being in a society where they felt they were different and were treated as inferior as they were African [4]. A questionnaire study by Pruitt [11] comprising 296 Sub-Saharan African students from nine American colleges further added that the students experienced homesickness while studying away from home [11]. It should however be noted that none of the above-mentioned studies specifically discussed the adjustment experiences of African health sciences students.

A qualitative study by Mattila et al. [8] investigating the experiences of international nurses in the Finnish education system indicated that the fourteen students of African and Asian origin had both positive and negative experiences with regard to adapting to the new system. Semistructured interviews were used to collect data where the students reported that while adjusting to Finnish health care system, they felt appreciated in the way that they were introduced to the clinical environment, staff members, patients, and the daily routines on the wards. Mattila et al. [8] asserted that the orientation helped the students adjust well to their new system as it gave them an understanding on how they could involve themselves in the activities in their new environment.

With regard to negative adjustment experiences, Mattila et al. [8] reported that one of the major hindrances for the students in adapting to the new the environment was the language. The students had a major problem communicating in Finnish, thereby making it difficult for them to adapt to Finnish hospital work culture [8]. While this study offers a reasonable account of the positive and negative experiences of Asian and African students as regards adjustment, it does not distinguish which experiences are specific to African students.

Omotosho [20] conducted a phenomenological study to explore the experiences of African nursing students. In adapting to the American culture, Omotosho [20] reported that the students experienced detachment from home, a place where they felt comfortable and secure. This study specifically utilized the term “stranger” to allude to the feelings of unfamiliarity, challenges with regard to being “different,” new cultural practices, and being in a new environment that the African students underwent in their adjustment journey.

### 4.2. Integrating

Studies have illuminated the experiences of African international students as they strive to assimilate into the existing culture and institutional environments [5, 21]. A qualitative study by Hyams-Ssekasi et al. [6] involving twenty-one postgraduate African students revealed that the students felt socially excluded and unwelcome by the local students. Isolation and feeling unwelcome impacted on the ability of the African students to work together with the local students, hence making the integrative process much more difficult [6]. The study further revealed that the African students discovered that studying at a university in the United Kingdom (UK) required a greater focus on self-directed learning and utilization of technology, aspects that they were previously not used to in their home countries, further complicating the integrative process. Interestingly, a study conducted by Manguvo [22] provided a slightly different way of looking at the integrative experiences of African students at an American university. The mixed methods study revealed that students who related more with fellow Africans tended to have more difficulties integrating than those who associated with the students from other racial backgrounds. Again, the above-mentioned studies do not specifically mention the experiences of African health sciences students.

In a study conducted by Sanner et al. [12] on the experiences of international students to whom English was a second language, eight Nigerian nursing students were interviewed. While trying to integrate into the academic and social components of the institution, the students reported feeling that they were not accepted. The researchers reported that the participants felt that American students, both Caucasian and African American, were not keen to listen to them, something that made their attempts to join study groups or nursing student organizations difficult [12]. These findings were corroborated by those in the study conducted by Constantine et al. [4] where African students were surprised by the discrimination that they faced from the African American students as they expected support from the African American group while trying to integrate into the majorly white environment. Overall, the two studies highlighted that the African students' difficulty in integrating may have been related to the perception in the host country that African students are intellectually inferior, a factor that was not aided by the apparent difficulties they faced with English. A study conducted by Shakya and Horsfall [23] in which another set of nursing students to whom English was a second language were interviewed highlighted the integrative challenges of the students in the Australian health education system. It was revealed that the students scored low grades in examinations and assignments as they misunderstood the requirements of the examiner or the assigned tasks. While the Shakya and Horsfall [23] study provided reasonable accounts of student experiences when striving to integrate into the Australian context, it is arguable that the study was conducted more than ten years earlier and the findings may not be current.

A cross-sectional study conducted by LaFleur [7] exploring the experiences of Nigerian nursing students in three American universities revealed that the students experienced isolation. The study in which 76 students were surveyed indicated that social support lacked for the students in their integrative journey in the new academic institutions.

### 4.3. Conditioning

Several studies on student experiences have illuminated the experiences of international students in their path to understanding how to operate in predominantly white environments. A study conducted by Khawaja and Stallman [24] with the aim of understanding the coping mechanisms of international students revealed that students experienced varied levels of stress, anxiety, and depression related to the idea of being in a new place and the mismatch between reality and previous expectations. The researchers however pointed out that with time the students learnt how to cope with the stress by accepting the reality and downgrading their expectations and through the use of other international students as support pillars [24]. The study also revealed that though most students had problems communicating in English on their arrival in Australia, they reported an improvement in their English communication skills due to work interactions. While the study offered detailed descriptions of student experiences, it is can be argued that the researchers made a superficial attempt at understanding the coping strategies of the minority students. Moreover, the experiences explored provided a generalist account on international student experiences instead of considering the distinct characteristics that exist between the different student racial groups.

A mixed methods study exploring the experiences and coping strategies of international medical students at an Australian university reported that the students went through a number of challenges while undertaking their studies [25]. This included difficulties with communication, overwhelming workloads, and financial difficulties. To overcome these obstacles, the participants focussed on the positives accruing from their experiences and the prestige associated with being a medical doctor. They further learnt to survive in a culture where one had to ask for help if needed, a departure from their previous setting where help was offered even without request [25]. While this study offered reasonable accounts of international medical students' experiences, the researchers point out that only third- and fourth-year students participated, and it is again not possible to identify experiences specific to African medical students.

Mentoring, advising, and the interactions between faculty and students have been highlighted as some essential factors in student success. Several studies investigating the experiences of minority students reveal that many students had major issues with the mentoring processes available to them. A study conducted by Nebedum-Ezeh [10] in the United States in which ten African students filled a questionnaire and were interviewed revealed that the students felt at loss for not having an African staff member involved in the running and administration of the affairs of the African students because the African staff member could easily connect to them and understand their plight. That notwithstanding, the students conditioned themselves to cope with the lack of support from people they could consider their own by forming strong bonds between each other and providing support to each other whenever needed [10]. The above findings could perhaps allude to findings in the study by Manguvo [22] in which African students rarely sought professional help in times of rife but instead turned to help groups formed by their fellow Africans for assistance.

On the contrary, in the study conducted by Shakya and Horsfall [23] the students pointed to the support they received from faculty as being instrumental in their struggle through nursing school. It is revealed that the students realised that the only way to succeed was to constantly seek counsel from their lecturers, who were more than willing to assist, hence having the ability to cope in the Australian education system.

## 5. Discussion

The analysis has provided insight into the experiences of African students as they try to adjust, integrate, and condition themselves to their new environments, dominated by white culture. The analysis brought to the fore the lived experiences of African students from the day when they started higher education to the time when they learnt how things were done and the tactics needed to survive. It was evident that adjusting to the new environment posed many challenges for African students, as they “felt different” and encountered language difficulties, which led to feelings of inferiority. Again, there was often the need to align one's expectations to a reasonable level, an aspect that proved to be a challenge to many students, as seen.

The integrative process was not easy either, as the students felt isolated and discriminated against. It was therefore imperative for the students to develop mechanisms that helped them to survive and cope with the challenges that their new environment “threw” at them, hence conditioning. Importantly, it was revealed that African students used each other as support systems while studying in predominantly white environments. Summarily, Osikomaiya [26] pointed out that the challenges that African students faced in their new learning environments enabled them to become resilient to racial stereotyping, further developing effective coping mechanisms that moved them towards growth and uptake of the numerous opportunities that the United States provides.

While a considerable number of studies published in the last 20 years reported on the experiences faced by various African students in predominantly white environments, it was revealed that only six papers directly explored the experiences of African health sciences students. Even so, the research has been carried out mostly in the American context. While some will argue that there would not be major differences between the experiences of African students, the argument is contestable. African health sciences mainly work in clinical areas and their interaction with patients could elicit different experiences. The only way of knowing is conducting research, exclusively with African health sciences students in clinical placement areas. More research detailing the adjustment, integrative, and conditioning processes of African student in non-American contexts is warranted due to the large number of African students seeking education in other predominantly white countries.

## 6. Implications and Recommendations

The literature analysis highlighted the challenges that African higher education students in predominantly white environments encountered. Higher education stakeholders have a task to ensure a level playing field for both majority and minority (African) population students. This could range from providing exhaustive preenrolment briefings where the students are aligned with the realities existing in the host countries, to educating the majority of students on minority (African) issues. It is evident that after examination of the past twenty years of literature, there has been some progress but further work is needed to enhance the learning of African students in developed countries studying in the health sciences.

## 7. Conclusion

This paper provided insight into the experiences of black African health sciences students in predominantly white environments. The dearth of information on the experiences of black African health sciences students was evident. Implications for higher education policy makers were forwarded and recommendations for possible research areas have been advanced.

## Figures and Tables

**Table 1:** Key articles.

Article	Author/s	Sample group	Methodology	Key results
African Women Pursuing Graduate Studies in the Sciences: Racism, Gender Bias, and Third World Marginality	Beoku-Betts [1]	15 African women scientists	Qualitative	(i) Gender bias (ii) Marginalization (iii) Racism (iv) Negative perception of African culture

Acculturative Experiences of Black-African International Students	Boafo-Arthur [3]	African students	Qualitative	(i) Prejudice and discrimination (ii) Social isolation (iii) Separation from family and friends (iv) Financial concerns

Examining the Cultural Adjustment Experiences of African International College Students: A Qualitative Analysis	Constantine et al. [4]	12 African students from 3 countries	Qualitative	(i) Varying pre- & postsojourn perceptions about the US (ii) Difficult cultural adjustment

Pre- and Post-Migration Attitudes among Ghanaian International Students Living in the United States: A Study of Acculturation and Psychological Well-Being	Fischer [5]	8 Ghanaian students	Qualitative (phenomenological)	(i) Discrimination (ii) Cultural difficulties (iii) Academic stress

International Education in the United Kingdom: The Challenges of the Golden Opportunity for Black-African Students	Hyams-Ssekasi et al. [6]	21 postgraduate African students	Qualitative (empirical) study	(i) Financial problems (ii) Disillusionment about value of international education

Acculturation, Social Support, and Self-Esteem as Predictors of Mental Health among Foreign Students: A Study of Nigerian Nursing Students	LaFleur [7]	76 Nigerian nursing students	Descriptive cross-sectional	(i) Isolation (ii) Low self-esteem

International Student Nurses' Experiences of Clinical Practice in the Finnish Health Care System	Mattila et al. [8]	14 African and Asian nursing students	Qualitative	(i) Appreciative orientation (ii) Sense of belonging (iii) Language barrier

Black African International Adult Students' Experiences in Higher Education: A Qualitative Study	Mwaura [9]	13 African students	Qualitative (phenomenological)	(i) Prejudice (ii) Isolation (iii) Financial constraints

An Examination of the Experiences and Coping Strategies of African Students at Predominantly White Institutions of Higher Education in the United States	Nebedum-Ezeh [10]	10 African students	Qualitative	(i) Lack of support from African staff members (ii) Resilience

The Adaptation of African Students to American Society	Pruitt [11]	296 Sub-Saharan African students	Questionnaire study	(i) Home sickness (ii) Discrimination (iii) Depression (iv) Irritability

The Experiences of International Nursing Students in a Baccalaureate Nursing Program	Sanner et al. [12]	8 Nigerian female nursing students	Qualitative	(i) Resilience (ii) Social isolation
